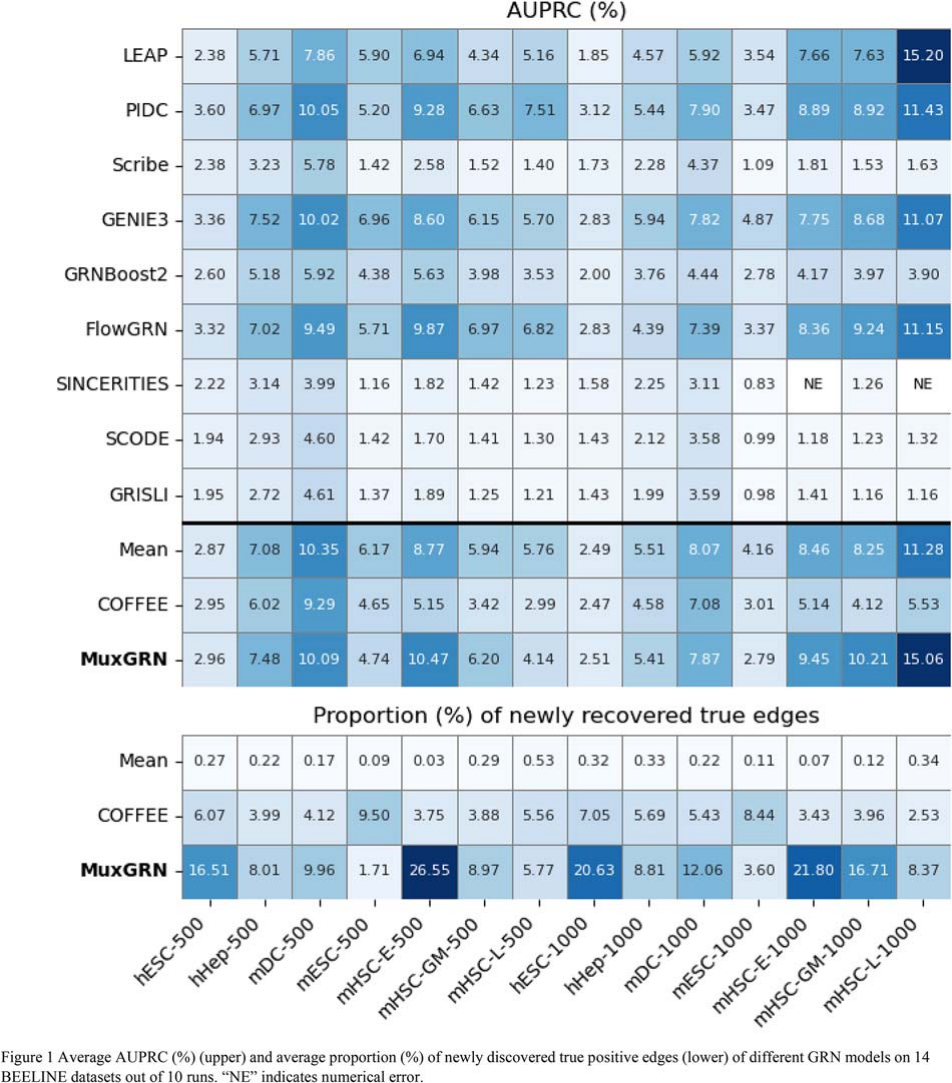# Subject section Aggregating GRNs via multiplex link prediction

**DOI:** 10.1093/bib/bbaf631.012

**Published:** 2025-12-12

**Authors:** Tsz Pan Tong, Jun Pang

**Affiliations:** Department of Computer Science, University of Luxembourg, 6 avenue de la Fonte, Esch-sur-Alzette 4364, Luxembourg; Institute for Advanced Studies, University of Luxembourg, 2 place de l'Université, Esch-sur-Alzette 4365, Luxembourg; Department of Computer Science, University of Luxembourg, 6 avenue de la Fonte, Esch-sur-Alzette 4364, Luxembourg; Institute for Advanced Studies, University of Luxembourg, 2 place de l'Université, Esch-sur-Alzette 4365, Luxembourg

## Abstract

Gene regulatory networks (GRNs) are essential in studying cell differentiation and development. However, existing GRN inference models often disregard the network nature of GRNs, undermining the potential for GRN inference from scRNA-seq data. Popular GRN models such as PIDC and GENIE3 only consider at most triplet interactions among genes, significantly underestimating the regulation complexity in real biological systems. To showcase the importance of the network structure in GRNs, we proposed a novel GRN aggregation method, MuxGRN, which leverages multiplex link prediction to aggregate GRNs inferred from various state-of-the-art models and extracts the information propagated within different layers of GRNs. We tested MuxGRN on 14 real scRNA-seq datasets aggregated from 9 GRN models and compared it with 2 baseline edge-level aggregation methods. Our evaluation shows that MuxGRN achieves a top-tier averaged AUPRC among individual GRN models and discovers up to 26.55% new true positive edges, significantly outperforming baselines. This highlights the potential of link prediction in GRN aggregation for mining weak signals from inferred GRNs, and suggests that future development of GRN models should emphasize the network nature of GRNs.

**Introduction:**

Gene regulatory networks (GRNs) are a core component in Systems Biology, capturing the complex interactions among genes that govern cell development and differentiation. Connected network modules are essential in gene regulatory analysis. Previous research shows that network motifs, such as feedforward loops and single-input modules, are meaningful functional units in GRN. From the perspective of information diffusion, regulations among genes are not isolated but propagated through the cascade in the network.

However, existing GRN inference methods often overlook the intrinsic network nature of GRNs and focus only on pairwise or triplet interactions among genes. Popular methods such as PIDC decompose the interaction up to three genes, and GENIE3 only quantifies the contribution of one gene to reconstruct the expression of the target gene. To capture the cascade of gene regulations, a dynamic model that propagates information is required to capture the temporal evolution of gene expression. Ordinary differential equation (ODE) methods such as SCODE use ODE to model such dynamics, but their performances are not competitive compared to state-of-the-art (SOTA) methods [1] since existing ODE models cannot tackle thousands of genes. However, the ODE methods’ weakness should not rule out the importance of GRN network structures.

To demonstrate the importance of GRN as a network, we proposed a novel GRN aggregation method, MuxGRN, which leverages variational graph autoencoder [2] (VGAE) to aggregate noisy GRNs inferred by various SOTA methods and discover new edges from information propagation within different layers of GRN. Different GRNs from GRN models can be viewed as a multiplex network, where each layer represents a GRN inferred by a specific method. We use CompGCN [3] to extract weak signals from different layers of GRNs and use the VGAE [2] framework to predict missing edges in a self-supervised manner. Our evaluation of 14 experimental data sets on the BEELINE benchmark [1] shows that MuxGRN can reach a level of performance similar to the best individual GRN model, of which up to 26.55% edges are new true positive edges. Our method outperforms two baseline edge-level aggregation methods, showcasing the potential of GRN aggregation via link prediction and a call for network-aware GRN inference.

**Related work:**

COFFEE [4] proposed using the Borda count for GRN aggregation, but the Borda count is an edge-level aggregation and does not capture the characteristics of the GRN network. GENECI [5] and Fujii et al. [6] aim at finding the optimal weight of GRN that maximizes some topological measures of the network. However, weighted-averaging GRNs have a low degree of freedom, and reweighting the GRN to improve network topology does not modify the relative edge strengths. Finally, methods such as EnsInfer [7] and EnGRaiN [8] train a meta-model using GRNs as input and part of the ground truth edges as labels to predict the rest of the ground truth, which is biased towards the ground truth and exposed to the risk of incorrect edges in the ground truth. Thus, a network-aware GRN aggregation method that can discover new edges without relying on the ground truth is still missing.

**Methodology:**

Our method, MuxGRN, aggregates GRNs inferred from various SOTA methods by multiplex link prediction. We deploy VGAE [2] to learn the node embeddings during the reconstruction of the multiplex network in a self-supervised manner. A multi-relational graph neural network model, CompGCN [3], is used as an encoder to extract weak signals from different layers of GRNs and compute node embeddings. The standard inner product decoder is used to reconstruct the multiplex network from the node embeddings, and new edges in the reconstructed network are considered as potential new regulations among genes. Detailed implementation is provided in our code repository https://github.com/1250326/MuxGRN.

**Evaluation:**

We evaluated MuxGRN on the BEELINE benchmark [1], which contains 7 sets of experimental scRNA-seq datasets for human and mouse biological systems. The top 500 (1000) high-variance genes and their transcription factors are selected from each dataset, resulting in 14 datasets in total with 501–1783 genes on 2000 cells. We used the STRING database as ground-truth GRNs, with 3220–85,424 edges and 0.92%–4.19% density. AUPRC is used as an evaluation metric to measure the model’s precision and discrimination ability. We also computed the proportion of newly discovered true positive edges apart from edges in the union of all individual GRN models, normalized by the best individual GRN models for aggregation baselines to show the ability of finding new edges.

We selected 9 SOTA GRN inference methods. GRNs are inferred using the default parameters in BEELINE, then aggregated by MuxGRN and two edge-level aggregation baselines, COFFEE [4] and Mean. COFFEE uses Borda count to compute the rank of each edge in each GRN and averages the ranks across different GRNs. In addition, we also designed a mean aggregation to compute the z-score of the edge weights in each GRN to denoise each GRN following the idea of CLR, and then average the z-scores across different GRNs.

**Limitation:**

Despite promising results, our method has several limitations. First, the STRING ground truth is incomplete and biased towards well-studied genes, which may affect the evaluation of GRN models. Besides, the STRING database also lacks directionality and signs of regulations, which are essential in GRNs. Second, our method cannot predict the directionality of regulations, which can be addressed using a biaffine decoder. Future work focuses on solving these limitations.

**Acknowledgments:**

Authors Tsz Pan Tong and Jun Pang acknowledge financial support from the Institute for Advanced Studies of the University of Luxembourg through an Audacity Grant (AUDACITY-2021). This work was also supported by the Luxembourg National Research Fund under the grant agreement INTER/NCN/24/18732364/EdgeCR.

**References:**

1. Pratapa A., Jalihal A.P., Law J.N., Bharadwaj A., and Murali T.M.. ‘Benchmarking algorithms for gene regulatory network inference from single-cell transcriptomic data.’ Nature Methods 2020;17(2):147–154.

2. Kipf T.N. and Welling M.. ‘Variational graph auto-encoders.’ arXiv preprint arXiv:1611.07308, 2016.

3. Vashishth S., Sanyal S., Nitin V., and Talukdar P.. ‘Composition-based multi-relational graph convolutional networks.’ In 8th International Conference on Learning Representations, 2020.

4. Lodi M.K., Chernikov A., and Ghosh P.. ‘COFFEE: consensus single cell-type specific inference for gene regulatory networks.’ Briefings in Bioinformatics 2024;25(6):bbae457.

5. Segura-Ortiz A., García-Nieto J., Aldana-Montes J.F., and Navas-Delgado I.. ‘GENECI: a novel evolutionary machine learning consensus-based approach for the inference of gene regulatory networks.’ Computers in Biology and Medicine 2023;155:106653.

6. Fujii C., Kuwahara H., Yu G., Guo L., and Gao X.. ‘Learning gene regulatory networks from gene expression data using weighted consensus.’ Neurocomputing 2017;220:23–33.

7. Shen B., Coruzzi G., and Shasha D.. ‘EnsInfer: a simple ensemble approach to network inference outperforms any single method.’ BMC Bioinformatics 2023;24(1):114.

8. Aluru M., Shrivastava H., Chockalingam S.P., Shivakumar S., and Aluru S.. ‘EnGRaiN: a supervised ensemble learning method for recovery of large-scale gene regulatory networks.’ Bioinformatics 2022;38(5):1312–1319.